# Concurrent Bacteremia and Severe Fever With Thrombocytopenia Syndrome: A Report of Two Cases

**DOI:** 10.7759/cureus.75408

**Published:** 2024-12-09

**Authors:** Yoshifumi Kubota, Hiroyuki Hiu, Yukiko Masuda, Chikaaki Nakamichi

**Affiliations:** 1 Department of Emergency and Critical Care Medicine, National Hospital Organization Nagasaki Medical Center, Nagasaki, JPN

**Keywords:** bacteremia, bacterial translocation (bt), concurrent bacteremia, gastrointestinal symptom, intestinal mucosal injury, severe fever with thrombocytopenia syndrome

## Abstract

Severe fever with thrombocytopenia syndrome (SFTS) is a tick-borne viral hemorrhagic fever caused by the severe fever with thrombocytopenia syndrome virus (SFTSV). This virus, which is transmitted through ticks, is prevalent in Asian countries, including Japan. This report describes two rare cases of SFTS with concurrent bacteremia. In the first case, a 73-year-old Japanese man presented with fever and coagulopathy and subsequently developed oral mucosal hemorrhage and bloody stools. Initial tests showed thrombocytopenia and leukopenia. Blood cultures on admission revealed *Enterococcus faecalis *bacteremia, and SFTSV co-infection was subsequently confirmed by polymerase chain reaction (PCR). He was treated with vancomycin, followed by ampicillin, and recovered. In the second case, a 70-year-old Japanese man presented with fever and diarrhea and subsequently developed renal impairment and thrombocytopenia. Blood cultures on admission revealed *Escherichia coli *bacteremia, and SFTSV co-infection was subsequently confirmed by PCR. He was treated with ceftriaxone, followed by ampicillin, and recovered. We conducted a literature search to look for evidence of a link between SFTS and bacteremia. Further epidemiological studies on the association between SFTS and bacteremia are warranted to provide an understanding of the pathogenic mechanisms.

## Introduction

Severe fever with thrombocytopenia syndrome (SFTS), a tick-borne viral hemorrhagic fever caused by severe fever with thrombocytopenia syndrome virus (SFTSV), was first reported in China in 2011 [1] and has subsequently been reported in several Southeast Asian countries [2]. Approximately 100 cases are reported annually in western Japan. Clinical symptoms of SFTS include fever, which is observed in nearly all cases, accompanied by symptoms such as headache, generalized fatigue, and gastrointestinal complaints, including vomiting, abdominal pain, and diarrhea [3]. Diagnosis of SFTS is performed using virus culture, polymerase chain reaction (PCR), and the detection of specific antibodies to SFTSV [3]. In Japan, testing for SFTSV is performed by laboratories in local public health institutes using PCR and is generally performed only on weekdays. No specific treatment is available for SFTS; therefore, management primarily involves providing supportive care [3]. A retrospective study conducted in Japan revealed a 27% case fatality rate [4].

Co-infections with Aspergillus and with bacterial pathogens have been reported in SFTS [5]. However, cases of SFTS with concurrent bacteremia are uncommon, with only a few cases reported in the literature. Herein, we describe two cases of SFTS with bacteremia and discuss the evidence of an association between SFTS and bacteremia, specifically bacteremia of gastrointestinal origin, and potential pathogenic mechanisms.

## Case presentation

Case 1

A 73-year-old Japanese man was admitted to our tertiary care hospital with a fever and signs of coagulopathy. He had a history of outdoor work, including brush clearing. He had developed a fever and general malaise four days before admission and had sought medical care at another hospital two days before admission. After admission to the first hospital, he developed oral mucosal hemorrhage and bloody stools. Blood tests revealed worsening thrombocytopenia; therefore, he was transferred to our hospital.

On admission to our hospital, his vital signs were as follows: blood pressure of 148/80 mmHg; pulse rate of 77 beats/min; respiratory rate of 22 breaths/min; SpO_2 _of 97% breathing room air; Glasgow Coma Scale (GCS) score of E3V4M6; and body temperature of 37.9°C. Physical examination revealed bleeding from the oral mucosa and purpura on the extremities. Blood tests revealed leukopenia and thrombocytopenia and elevated levels of aspartate aminotransferase, alanine transaminase, creatinine, and ferritin (Table 1).

**Table 1:** Blood test results of each patient on admission to our hospital APTT: activated partial thromboplastin time; FDP: fibrin/fibrinogen degradation products; NA: not available; PT-INR: prothrombin time-international normalized ratio Bold values indicate those outside the normal range

Test (units)	Normal range	Case 1	Case 2
White blood cell count (cells/µL)	3300-8600	3400	1900
Hemoglobin (g/dL)	13.7-16.8	16.0	14.6
Hematocrit (%)	50.1-40.7	48.3	40.2
Platelet count (/µL)	158000-348000	51000	56000
Total protein (g/dL)	6.6-8.1	6.2	5.9
Albumin (g/dL)	4.1-5.1	3.1	3.1
Aspartate aminotransferase (U/L)	13-30	243	173
Alanine transaminase (U/L)	10-42	126	57
Lactate dehydrogenase (U/L)	124-222	851	669
Alkaline phosphatase (U/L)	38-113	99	68
γ-glutamyltransferase (U/L)	13-64	168	47
Total bilirubin (mg/dL)	0.4-1.5	0.6	0.4
Blood urea nitrogen (mg/dL)	8.0-20.0	17.8	66.9
Creatinine (mg/dL)	0.65-1.07	1.19	3.05
Sodium (mEq/L)	138-145	137	130
Potassium (mEq/L)	3.6-4.8	4.3	3.3
Chlorine (mEq/L)	101-108	107	100
C-reactive protein (mg/dL)	<0.14	0.04	0.24
Procalcitonin (ng/mL)	<0.10	0.09	3.29
Ferritin (ng/mL)	20-200	5972	7180
PT-INR	0.8-1.1	0.97	1.07
APTT (s)	24.3-34.6	47.1	41.8
FDP (μg/mL)	≤5	3.3	21.7
D-dimer (μg/mL)	≤1	1.4	NA

Whole-body computed tomography (CT) revealed no abnormalities.

He was admitted to the intensive care unit (ICU) with leukopenia, thrombocytopenia, and disseminated intravascular coagulation (DIC). The possibility of bacterial or rickettsial infection with organ failure was considered; therefore, blood and urine samples were collected and sent for culture, and empiric antimicrobial therapy with ceftriaxone and minocycline was initiated. Based on his clinical presentation and laboratory findings of leukopenia, thrombocytopenia, and elevated transaminase and ferritin levels, the possibility of SFTS was also considered, and a whole blood sample was sent to the local public health institute for SFTSV testing. On day 2 of hospitalization, gram-positive cocci were observed in both sets of blood culture samples collected on admission. Vancomycin therapy was initiated in addition to continuing the ceftriaxone and minocycline initiated on admission. Repeat blood cultures were conducted to confirm the clearance of the bacteremia. The culture of the urine sample collected on admission revealed *Citrobacter koseri*. On day 6 of hospitalization, the local public health institute reported that the blood sample had tested positive for SFTSV on PCR, confirming the diagnosis of SFTS. The gram-positive cocci in the admission blood culture samples were subsequently identified as *Enterococcus faecalis*, and vancomycin was switched to ampicillin based on the antimicrobial susceptibility testing results (Table 2).

**Table 2:** Results of antimicrobial susceptibility testing of the bacteria isolated from the bloodstream of each patient MIC: minimum inhibitory concentration; NA: not applicable; S: susceptible; R: resistant

Antimicrobials	Case 1: *Enterococcus faecalis*		Case 2: *Escherichia coli*	
	MIC (μg/mL)	Interpretation	MIC (μg/mL)	Interpretation
Penicillin G	4	S	4	S
Ampicillin	≤2	S	≤2	S
Piperacillin	NA	NA	≤4	S
Ampicillin/sulbactam	≤2	S	≤2	S
Piperacillin/tazobactam	NA	NA	≤4	S
Cefazolin	NA	NA	≤4	NA
Cefixime	≥64	R	NA	NA
Ceftazidime	NA	NA	≤1	S
Ceftriaxone	NA	NA	≤1	S
Cefepime	NA	NA	≤1	S
Imipenem/cilastatin	≤1	S	≤0.25	S
Meropenem	NA	NA	≤0.25	S
Gentamicin	NA	NA	≤1	S
Amikacin	NA	NA	≤2	S
Clindamycin	≤8	R	NA	NA
Minocycline	≥16	R	≤1	S
Vancomycin	1	S	NA	NA
Teicoplanin	≤0.5	S	NA	NA
Ciprofloxacin	NA	NA	≤0.25	S
Levofloxacin	0.5	S	≤0.12	S
Sulfamethoxazole-trimethoprim	≤10	R	NA	NA

The results of the repeat blood culture of samples collected on day 2 of hospitalization were negative. Serial blood tests performed during the hospitalization revealed a gradual resolution of leukopenia and a steady increase in the platelet count.

The patient had a favorable clinical course and was transferred from the ICU to the general ward on day 4 of hospitalization. He received a 14-day course of antimicrobial therapy and was discharged on day 18 of hospitalization.

Case 2

A 70-year-old Japanese man who lived in a shed with farm equipment was admitted to our hospital with a fever. He had developed a fever and diarrhea eight days before admission and had sought medical care at another hospital two days before admission. His laboratory test results at that time showed worsening renal impairment and thrombocytopenia; therefore, he was transferred to our hospital.

On admission to our hospital, his vital signs were as follows: blood pressure of 112/62 mmHg; pulse rate of 76 beats/min; respiratory rate of 24 breaths/min; SpO_2 _of 98% breathing room air; GCS score of E4V4M6; and body temperature of 37.9°C. The findings of the physical examination were unremarkable. Blood tests revealed leukopenia and thrombocytopenia and elevated levels of aspartate aminotransferase, alanine transaminase, creatinine, and ferritin (Table 1). Whole-body CT showed no abnormalities.

He was admitted to the ICU with leukopenia, thrombocytopenia, and DIC. After submitting blood and urine samples for culture, empiric antimicrobial therapy was initiated with ceftriaxone and minocycline, as in Case 1. On day 2 of hospitalization, gram-negative bacilli were observed in one of the two sets of blood culture samples collected on admission, but the urine culture was negative. Ceftriaxone was continued. Repeat blood cultures were conducted to confirm the clearance of the bacteremia. Based on his clinical presentation and laboratory findings of leukopenia, thrombocytopenia, and elevated transaminase and ferritin levels, the possibility of SFTS was considered, and a whole blood sample was sent to the local public health institute for SFTSV testing on day 4 of hospitalization. The gram-negative bacilli in the admission blood culture sample were subsequently identified as *Escherichia coli*; therefore, the antibiotic was switched from ceftriaxone to ampicillin based on the susceptibility testing results (Table 2). On day 6 of hospitalization, the local public health institute reported that the blood sample had tested positive for SFTSV on PCR, confirming the diagnosis of SFTS.

The results of the repeat blood culture of samples collected on day 5 of hospitalization were negative. Serial blood tests showed a gradual resolution of leukopenia and an improvement in kidney function during hospitalization. The platelet count reached its lowest value of 37000/μL on day 3 of hospitalization and subsequently increased.

The patient had a favorable clinical course and was discharged from the ICU on day 6 of hospitalization. He received a 14-day course of antimicrobial therapy and was transferred back to the original hospital to continue rehabilitation on day 17 of hospitalization.

## Discussion

These two cases of SFTS with bacteremia both had favorable outcomes. Few cases of SFTS with bacteremia have been reported in the medical literature. One report described a case of SFTS with *E. coli* bacteremia [6], and another report described a fatal case of SFTS with *Pasteurella multocida* bacteremia in a patient who owned multiple cats [7]. A retrospective study of 1201 hospitalized patients with SFTS reported community-onset bacteremia in 2.3% of cases [8]. Another retrospective study of 109 patients with SFTS-related encephalitis/encephalopathy reported bacteremia in 11% of cases, with causes including coagulase-negative *Staphylococcus*, *Streptococcus* spp., *E. coli*, *Enterobacter cloacae*, and *Pseudomonas aeruginosa* [9].

In both of our patients, the apparent source of bacteremia was gastrointestinal infection. One patient had enterococcal bacteremia, and the other had *E. coli* bacteremia. The usual sources of community-onset enterococcal and *E. coli* bacteremia are the urinary tract or intra-abdominal infection [10,11]. Urine culture did not indicate the presence of the bacteria as a source of the bacteremia in either case. Enterococcal bacteremia is sometimes associated with infective endocarditis [10]; however, transthoracic echocardiography and other investigations did not show any signs of infective endocarditis. Hence, intra-abdominal infection was the most likely source of bacteremia in both cases.

SFTSV infection has been reported to be associated with gastrointestinal mucosal disorders. Daisuke et al. [12] conducted a study using 18F-fluorodeoxyglucose (18F-FDG) positron emission tomography on mice infected with SFTSV, which revealed an abnormal accumulation of 18F-FDG in the intestinal tract. Additionally, SFTSV genes were detected in epithelial cells of the stomach and intestine, suggesting that SFTSV infection is associated with gastrointestinal disorders. Disruption of the gastrointestinal barrier may enhance bacterial translocation [13]. In our two patients, the only abnormal abdominal findings noted were diarrhea and bloody stools, suggesting that the bacteria may have entered the bloodstream through damaged intestinal mucosa.

SFTSV infection may be associated with alterations in the gut microbiota and an increase in the abundance of pathogenic bacteria. Honghai et al. [14] compared the gut microbiota of individuals diagnosed with SFTS and healthy controls using next-generation sequencing. Both groups resided in the same region, and individuals who had underlying gastrointestinal diseases or antibiotic use within the previous three months were excluded from the study. The study revealed that the phylum *Firmicutes* was significantly more abundant in individuals with SFTS than in healthy controls and that levels of the genera *Streptococcus*, *Lactobacillus*, and *Enterococcus* were elevated in the patients with SFTS. Additionally, bacteria from the phylum *Proteobacteria*, particularly from the family *Enterobacteriaceae*, were more abundant in individuals with SFTS, with a notable increase in the abundance of *Escherichia*. These findings suggest that pathogenic bacteria in genera such as *Streptococcus*, *Enterococcus*, and *Escherichia* may play a role in the pathogenesis of SFTS.

These findings suggest that patients with SFTS may be more susceptible to bacteremia originating from the gastrointestinal tract owing to SFTSV-induced injury to the mucosal barrier and a shift toward a more pathogenic enteric microbiota.

A retrospective observational study by Kutsuna et al. [15] reported that early antibiotic administration in hospitalized patients with SFTS had no effect on in-hospital mortality. However, in specific cases, such as patients with gastrointestinal symptoms, as observed in our two cases, early antibiotic therapy may offer potential clinical benefits.

Notably, cases of bacteremia involving non-enteric organisms, such as *P. multocida*, suggest that mechanisms other than alterations in the gut microbiota and intestinal mucosal damage may be involved. These findings highlight the need for further investigation into the mechanisms whereby SFTSV infection may increase susceptibility to bacteremia.

## Conclusions

These two cases of SFTS with concurrent bacteremia, of probable gastrointestinal origin, suggest that patients with SFTS who present with gastrointestinal symptoms may have an increased risk of bacteremia of enteric origin, particularly as their clinical condition worsens. Further, epidemiological studies are needed to determine the characteristics of patients with SFTS who develop concurrent bacteremia, the distribution of different types of bacteria, and possible sites of entry into the bloodstream. Such research might enhance our understanding of the underlying pathogenic mechanisms and enable early identification and treatment of individuals at risk of developing bacteremia as a complication of SFTS.
